# Simultaneous quantitative chiral analysis of four isomers by ultraviolet photodissociation mass spectrometry and artificial neural network

**DOI:** 10.3389/fchem.2023.1129671

**Published:** 2023-03-09

**Authors:** Yingying Shi, Ming Zhou, Min Kou, Kailin Zhang, Xianyi Zhang, Xianglei Kong

**Affiliations:** ^1^ State Key Laboratory of Elemento-Organic Chemistry, College of Chemistry, Nankai University, Tianjin, China; ^2^ School of Physics and Electronic Information, Anhui Normal University, Wuhu, China; ^3^ Life and Health Intelligent Research Institute, Tianjin University of Technology, Tianjin, China; ^4^ Tianjin Key Laboratory of Biosensing and Molecular Recognition, College of Chemistry, Nankai University, Tianjin, China; ^5^ Frontiers Science Center for New Organic Matter, College of Chemistry, Nankai University, Tianjin, China

**Keywords:** chiral analysis, multiple isomers, mass spectrometry, ultraviolet photodissociation, dipeptide

## Abstract

Although mass spectrometry (MS) has its unique advantages in speed, specificity and sensitivity, its application in quantitative chiral analysis aimed to determine the proportions of multiple chiral isomers is still a challenge. Herein, we present an artificial neural network (ANN) based approach for quantitatively analyzing multiple chiral isomers from their ultraviolet photodissociation mass spectra. Tripeptide of GYG and iodo-L-tyrosine have been applied as chiral references to fulfill the relative quantitative analysis of four chiral isomers of two dipeptides of ^*L/D*^His^*L/D*^Ala and ^*L/D*^Asp^*L/D*^Phe, respectively. The results show that the network can be well-trained with limited sets, and have a good performance in testing sets. This study shows the potential of the new method in rapid quantitative chiral analysis aimed at practical applications, with much room for improvement in the near future, including selecting better chiral references and improving machine learning methods.

## 1 Introduction

Chirality exists in various types of organic and biological molecules, including amino acids, carbohydrates, DNAs and proteins. The chiral isomers may have quite different interactions with their surroundings in living systems, resulting in different pharmacological activities (Nguyen et al., 2006). At the early ages, it was still believed that D-amino acids only occurred in microorganisms and some peptides. Due to the development of analytical methods in the past decades, the presence of D-amino acids in higher organisms, including human beings, is proven (Genchi, 2017). Thus, chiral analysis is very important for both fundamental and applied research. Various methods have been developed and applied for effective chiral analysis, including circular dichroism (CD) spectroscopy, nuclear magnetic resonance (NMR), and chromatographic methods, such as liquid chromatography (LC) or gas chromatography (GC) (Nguyen et al., 2006; Ward and Baker, 2008; Lanucara et al., 2014; Wenzel, 2018; Hu et al., 2020). It should be mentioned that the selection of suitable reagent or ligand to fulfill chiral analysis or to assign the absolute configuration of particular compounds is very important in such experiments (Müller et al., 2008; Scaramuzzo et al., 2013).

Since the ions of enantiomers (or chiral isomers) have the same m/z, mass spectrometry (MS) was considered as a blind method for chiral analysis in the early years. However, the rapid development of mass spectrometry methods, especially in the field of soft ionization, has undergone great changes (Tao and Cooks, 2003; Wu et al., 2012; Awad and El-Aneed, 2013; Yu and Yao, 2017; Han and Yao, 2020; Shi et al., 2020). In recent years, MS based chiral analysis methods have attracted widespread attention due to its unique advantages in speed, specificity and sensitivity. Differentiation of chiral isomers can be achieved by comparing the formation, dissociation or reaction behavior of the diastereomers in the gas phase, or their mobilities when combined with the technique of ion mobility (Tao and Cooks, 2003; Wu et al., 2012; Awad and El-Aneed, 2013; Lanucara et al., 2014; Yu and Yao, 2017; Han and Yao, 2020; Hu et al., 2020; Shi et al., 2020). The method of infrared multiple photon dissociation (IRMPD) and ultraviolet photodissociation (UVPD) spectroscopic methods, have also applied to the field and have shown their advantages in providing spectral and structural information of corresponding chiral complexes (Filippi et al., 2012; Liao et al., 2013; Fujihara et al., 2016; Lee et al., 2017; Ren et al., 2017; Fujihara and Okawa, 2018; Lee et al., 2018; Ma et al., 2018; Shi et al., 2019; Shi et al., 2020; Sun et al., 2020). Among these MS-based methods, UVPD has been demonstrated to be able to yield abundant radicals in fragmentation processes, thus provides a unique means for generating novel dissociation pathways in tandem mass spectrometry (Ly and Julian, 2009).

Although progresses have been achieved in qualitative chiral analysis, quantitative analysis aimed to determine the value of enantiomeric excess (ee) is more difficult and complicated than qualitative analysis aimed to recognition (Fujihara et al., 2017). On the other hand, most of the MS-based chiral analysis studies still focused on enantiomers with one chiral center. For the complicated compounds with multiple chiral centers, the recognition of the multiple isomers including both enantiomers and diastereomers in a single experiment will be more difficult, not to mention the quantitative analysis of these compounds. Among the D-amino acids, D-Ala is an unusual endogenous amino acid present in invertebrates and vertebrates, and its function in the mammalian nervous and endocrine systems is significant (Lee et al., 2020). And D-Asp is one of the major regulators of adult neurogenesis and plays an important role in the development of endocrine function (Genchi, 2017). In this paper, two dipeptides including L/D-Ala and L/D-Asp were selected here as the sample molecules to establish a new analytical method. Herein, we show that the quantitative analysis of four chiral isomers of dipeptides can be achieved by combining the methods of UVPD MS performed with a tunable UV laser and artificial neural network (ANN).

## 2 Experimental

The experimental setup has been described in our previous paper (Shi et al., 2019; Zhang et al., 2020). Briefly, a 7.0 T Fourier transform ion cyclotron resonance (FT ICR) mass spectrometer (IonSpec, Varian, Inc., Lake Forest, CA) was applied here, combined with one commercial UV-Vis tunable laser (NT-342C, EKSPLA, Lithuania). The laser was operated in normal mode in the range of 210–300 nm, with a typical output energy of 1–2 mJ/pulse in this experiment. The laser was introduced coaxially to the ICR cell through a CaF_2_ window and the irradiation time was set as 4s that was controlled though a mechanical shutter controller (SSH, Sigma-Koki, Tokyo, Japan).

Chiral dipeptides of ^*L/D*^His^*L/D*^Ala and ^*L/D*^Asp^*L/D*^Phe were ordered from Ontores and Shanghai Apeptide companies, respectively. And the chiral reference molecules of tripeptide GYG and 3-Iodo-L-tyrosine were brought from GL Bopchem (Shanghai) and DAMAS-BETA, respectively. Each sample was prepared in deionized water with a concentration of 10 mmol/L and then diluted to 1 mmol/L aqueous solutions with 49% methanol and 2% acetic acid before mixing. The solution of chiral reference was then mixed with the solution of dipeptide in a volume ratio of 1:1, and the later was prepared by premixing the 4 kinds of chiral isomers as designed. In the MS experiments, a Zspray electrospray ionization (ESI) source was applied with a probe biased at 3.6 kV. The complex ions were selected by the method of stored waveform inverse Fourier transform (SWFIT), followed by the UV irradiation (Cody et al., 1987; Shi et al., 2019). After the UVPD process, the product ions were detected and the mass spectrum was recorded. The wavelengths of the UV laser can be readily selected and changed with the commercial program provided by the laser manufactory.

**FIGURE 1:**
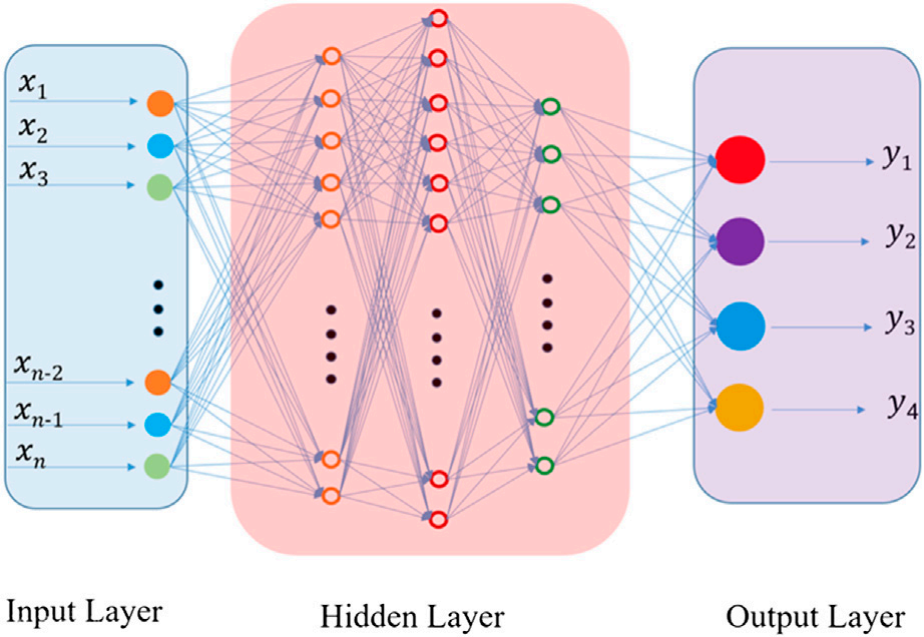
Illustration of the applied artificial neural network (ANN) model in this paper.

For the applied artificial neural network, a three-layered backpropagation (BP) network was designed with MATLAB mathematical software, in which sigmoid transfer function at hidden layer was used (Figure 1). The input variables were peak intensities of ions with specified m/z’s under different wavenumbers. And the corresponding molar ratios of the four chiral isomers were chosen as the target. The data sets were divided into training and test subsets, respectively.

## 3 Results and discussion

**FIGURE 2:**
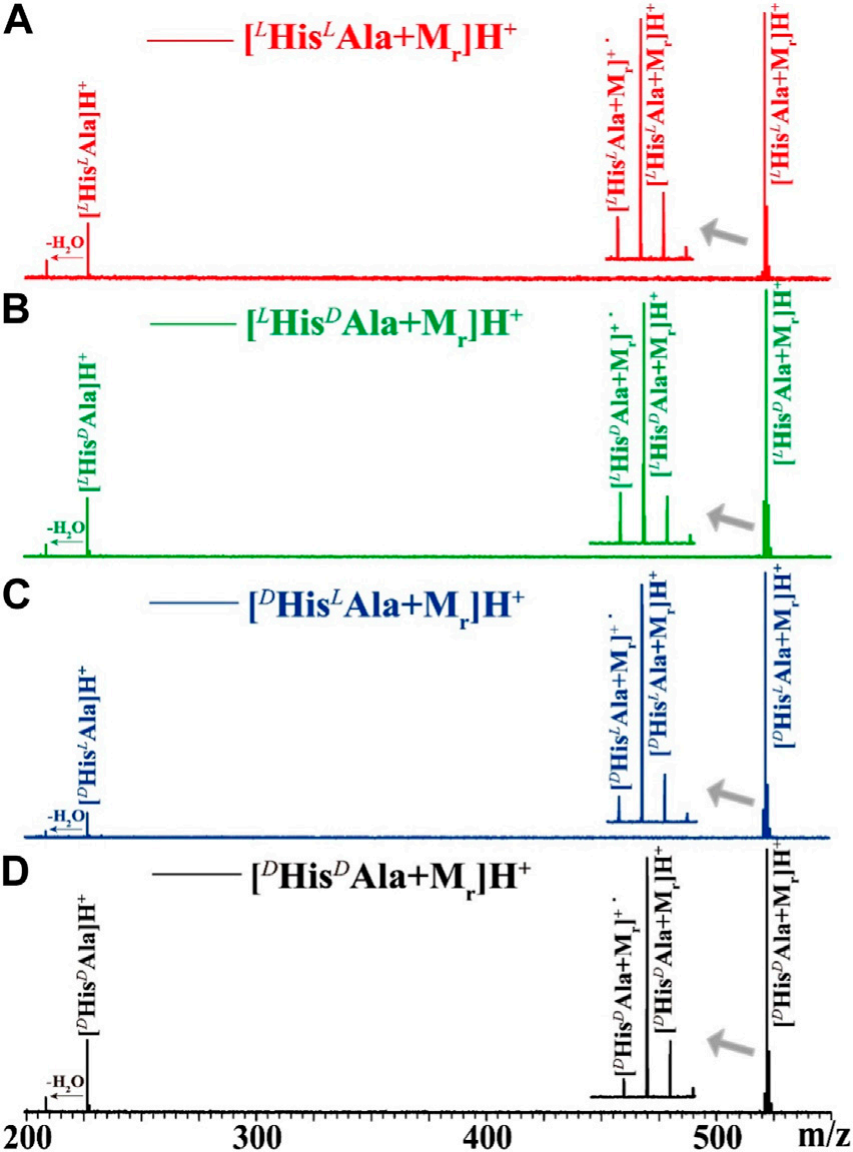
225 nm UV photodissociation mass spectra of **(A)** [^*L*^His^*L*^Ala + M_r_]H^+^, **(B)** [^*L*^His^*D*^Ala + M_r_]H^+^, **(C)** [^*D*^His^*L*^Ala + M_r_]H^+^, and **(D)** [^*D*^His^*D*^Ala + M_r_]H^+^, in which the M_r_ indicates the reference molecule of tripeptide GYG. Details of the mass spectra near the precursor ions are further shown in the insets.

**FIGURE 3:**
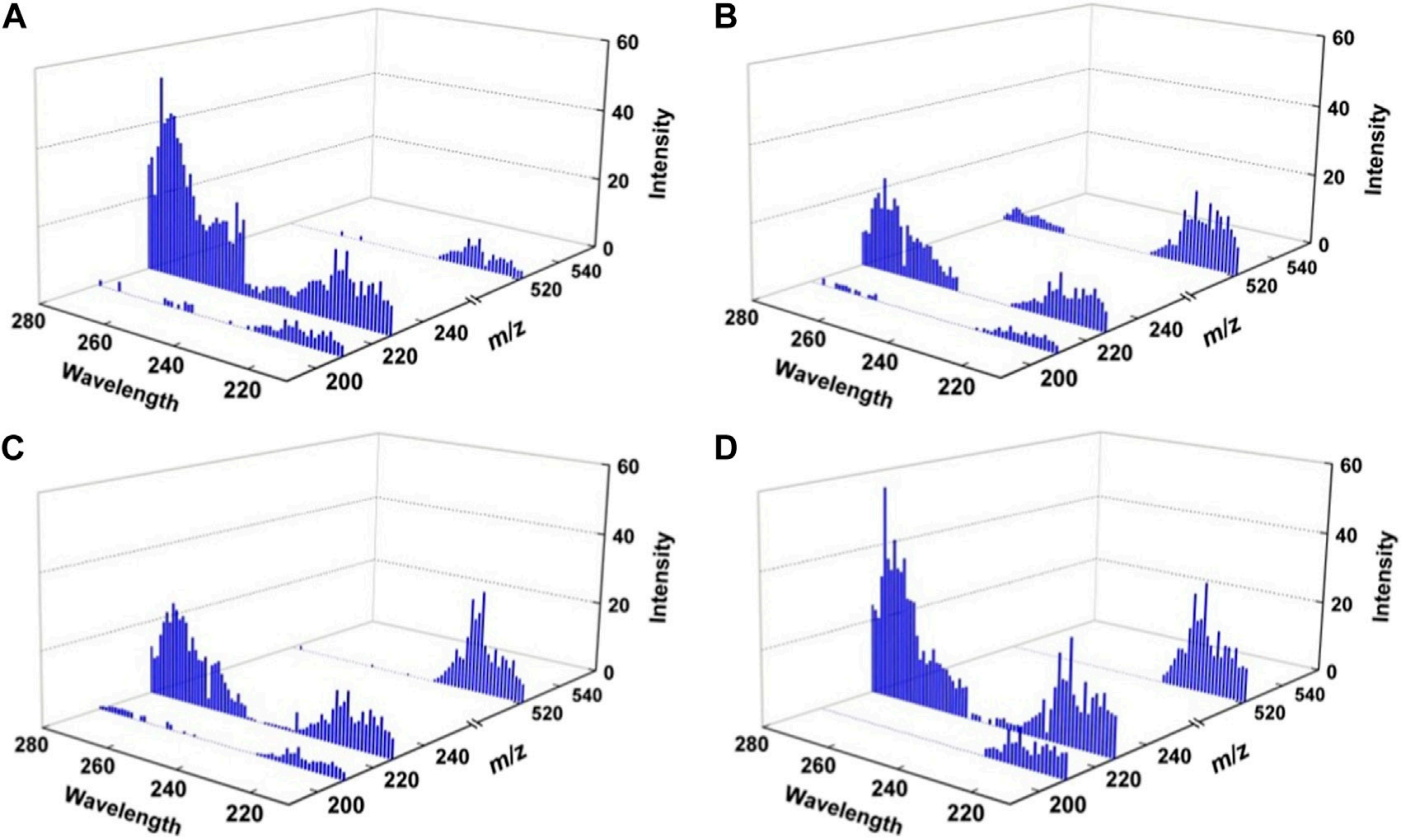
Three-dimensional UV photodissociation mass spectra of **(A)** [^*L*^His^*L*^Ala + M_r_]H^+^, **(B)** [^*L*^His^*D*^Ala + M_r_]H^+^, **(C)** [^*D*^His^*L*^Ala + M_r_]H^+^, and **(D)** [^*D*^His^*D*^Ala + M_r_]H^+^.

Considering the success of peptide as the ligand applied in the chiral analysis of short peptides (Tao, et al., 2001), the tripeptide of GYG (M_r_) is selected as a reference molecule for the four analytes of ^*L/D*^His^*L/D*^Ala. The complex ions of [HisAla + M_r_]H^+^ were generated by ESI from corresponding mixed solutions. The target ions were then selected and trapped in the FT ICR cell, followed by irradiation of UV laser at suitable wavelengths. As an example, the 225 nm UVPD mass spectra of the four complex ions are shown in Figure 2. The UVPD mass spectra are readily to be read. For all cases, three fragment ions can be found: the radical cations of [HisAla + M_r_]^+•^ formed by the loss of hydrogen atom, the protonated dipeptide ions of [HisAla]^+^ and their dehydrated forms. The relative intensities of the three fragment ions are kinds of different from each other, but can be hardly direct applied for chiral analysis for the four chiral compounds. The difference among them can be made clearer if three-dimensional dissociation mass spectra obtained under different UV wavelengths were applied (Figure 3).

**FIGURE 4:**
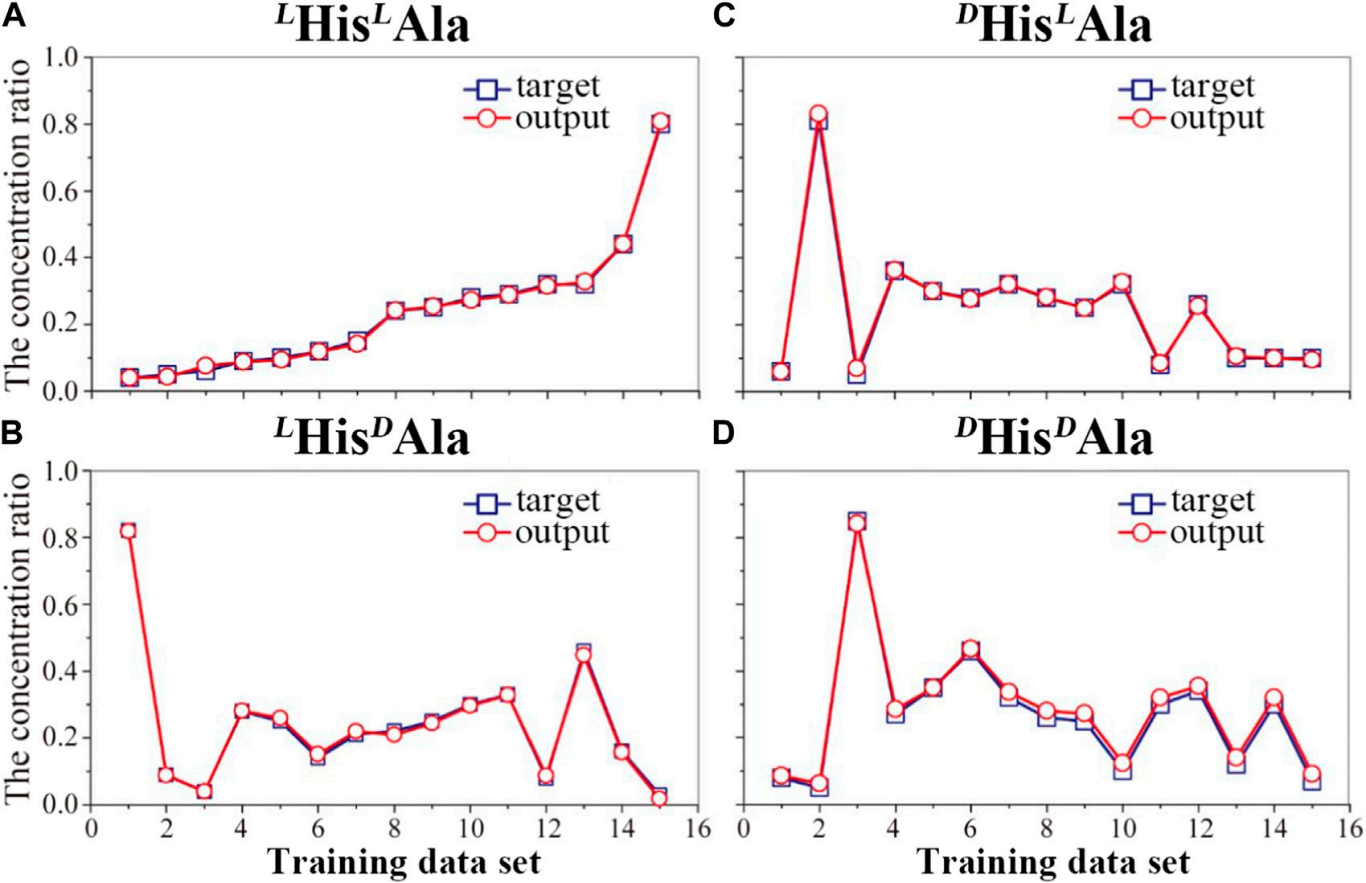
Trained results of the artificial neural network for the quantitative analysis of the four peptides: **(A)** ^*L*^His^*L*^Ala, **(B)** ^*L*^His^*D*^Ala, **(C)** ^*D*^His^*L*^Ala and **(D)** ^*D*^His/^*D*^Ala in mixture samples.

**FIGURE 5:**
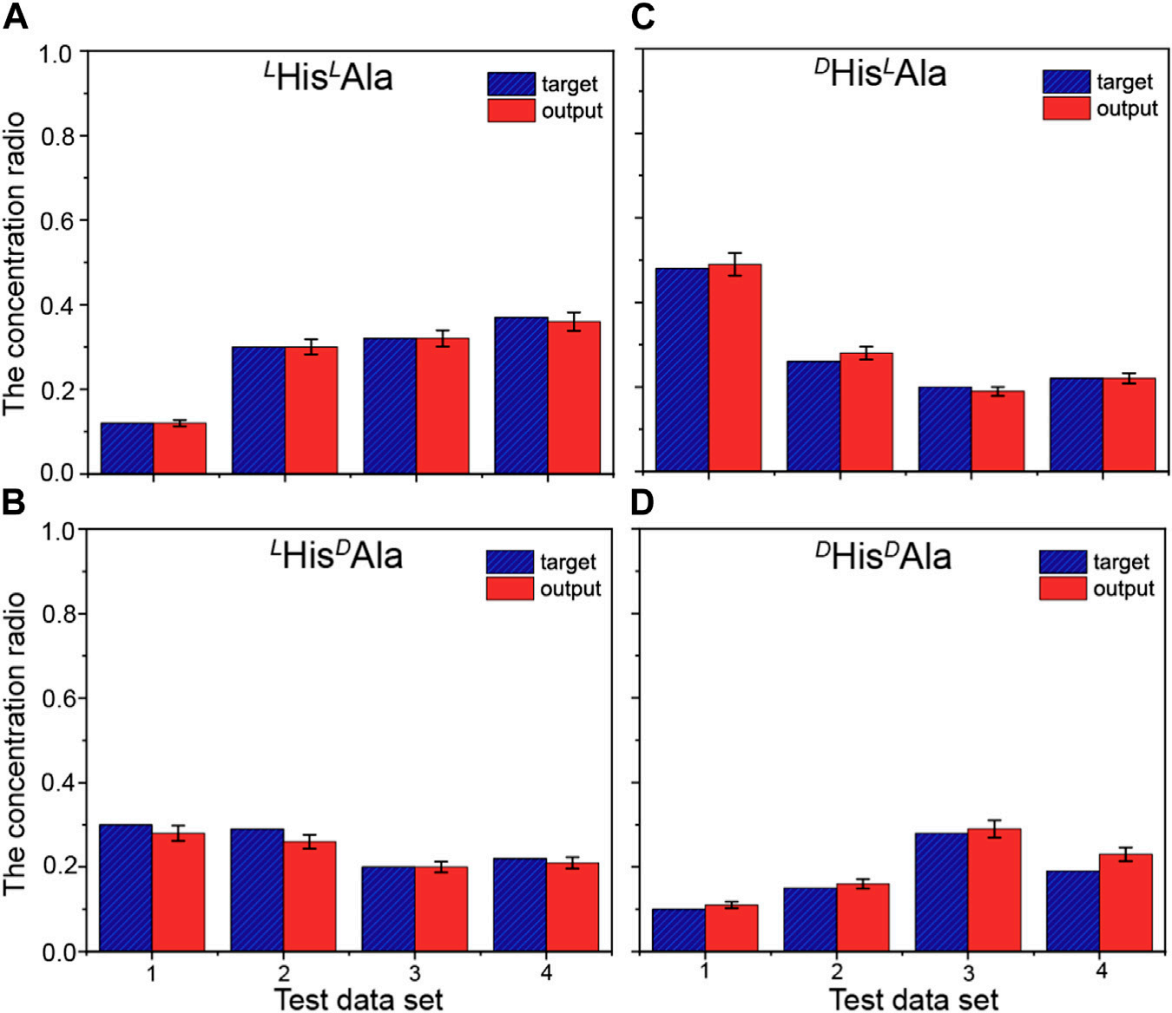
Comparisons between the true concentration ratios (blue striped columns) and predicted results (red columns) for the quantitative analysis of the four peptides: **(A)** ^*L*^His^*L*^Ala, **(B)** ^*L*^His^*D*^Ala, **(C)** ^*D*^His^*L*^Ala and **(D)** ^*D*^His/^*D*^Ala. The predictions were performed for four date sets, using the artificial neural network built based on the training set shown in Figure 4. The standard deviations of the predicted results based on three independent experimental data are less than 8%.

Although the difference, it is still too difficult to apply the method to quantitative analysis of these chiral isomers directly since the quantitative evaluation of the difference is difficult. In order to overcome the obstacle, the method of machine learning based on artificial neural network (ANN) was introduced here. To fulfill it, four wavelengths of 210, 213, 225 and 230 nm were selected here. A total of 19 mixed solutions with different proportions of the four chiral isomers were prepared and mixed with the reference molecule. The generated complex ions were further isolated and irradiated under the four different UV wavelengths. All the UVPD mass spectra were recorded. The data from the first 15 samples were applied as the training sets for the network, followed by training under supervision. Figure 4 shows the training results of the artificial neural network applied here (Figure 1). The error between the training output value and the real value is small, which indicates that the artificial neural network has been trained. To prevent possible over-fitting in the training process, two methods were applied here: 1) to design the training sets carefully and to ensure its proper population, 2) to adopt the early stopping method (Deng et al., 2015). After the training, the last four data sets are tested. And the results are shown in Figure 5. The predicted values are in good agreement with the real ones, and the standard deviations are less than 8%.

The most valuable variables in such an experiment are the distributions of fragment ions and how the distributions depend on the applied wavelengths. Thus, the selection of chiral ligands is important. For the second case, a small chiral reference of iodo-L-tyrosine is selected. The molecule has been previously studied in the gas phase. Ranka et al. have studied the radical rearrangement chemistry of the molecule using 193 nm UVPD mass spectrometry, IR ion spectroscopy and calculations, discovered that the high-energy radicals generated by UVPD engaged in following hydrogen/proton rearrangement (Ranka et al., 2018). The molecule has been also successfully applied as a good chiral reference for differentiation of enantiomeric pairs of amino acids and some pharmaceutically important drugs (Kumari et al., 2007; Karthikraj1 et al., 2012). Similarly, the 280 nm UVPD mass spectra of the four complex ions are shown in Supplementary Figure S1. The main fragment ions observed in the spectra include: the radical cations of [AspPhe + M_r_-I]H^+•^ formed by the loss of iodine atom, and its product of [AspPhe + M_r_-I]^+^, the protonated ions of [AspPhe]H^+^, its radical cations of [AspPhe]^+•^ and its dehydrated ions of [AspPhe-H_2_O]H^+^. By tuning the applied UV wavelength, the intensities of those fragment ions vary differently for the four chiral isomers. A similar artificial neural network described in the upper case has been built, and relative quantitative analysis of the multiple chiral isomers has been fulfilled using very limited training sets (10 samples), and the results are shown in the Supplementary Figures S2, S3.

## 4 Conclusion

In summary, we herein present an ANN approach for analyzing four chiral isomers from their UVPD mass spectra obtained under different wavelengths. Chiral reference molecules of tripeptides and iodo-L-tyrosine, have been successfully applied here to fulfill the relative quantitative analysis of four chiral isomers of dipeptides. The results show that the ANN can be well-trained with limited training sets, and have a quite good performance in testing sets. Meanwhile, the combination of the multiple UVPD mass spectra and ANN still has a lot of room for improvement in quantitative chiral analysis aimed at practical applications, such as searching better chiral references (Yu and Yao, 2017), finding possible derivatization methods (Will et al., 2021), and choosing more advanced machine learning methods.Fujihara and Maeda, 2017

## Data Availability

The raw data supporting the conclusion of this article will be made available by the authors, without undue reservation.
