# Supplementary material for: Simultaneous quantitative chiral analysis of four isomers by ultraviolet photodissociation mass spectrometry and artificial neural network
Source: Front Chem. 2023 Mar 9;11:1129671. doi: 10.3389/fchem.2023.1129671 (PMC10034024; doi:10.3389/fchem.2023.1129671)
Supplement: Supplementary file 1 [file DataSheet1.docx]

Supplementary Material

**Simultaneous Quantitative Chiral Analysis of Four Isomers by Ultraviolet Photodissociation Mass Spectrometry and Artificial Neural Network**

**Yingying Shi^1^, Ming Zhou^1,2^, Min Kou^1^, Kailin Zhang^3^, Xianyi Zhang^2^, Xianglei Kong ^1,4,5*^ **

^1^Elemento-organic Chemistry, Collage of Chemistry, Nankai University, Tianjin, 300071, China

^2^School of Physics and Electronic Information, Anhui Normal University, Wuhu, 241000, China

^3^Life and Health Intelligent Research Institute, Tianjin University of Technology, Tianjin 300384, China

^4^Tianjin Key Laboratory of Biosensing and Molecular Recognition, College of Chemistry, Nankai University, Tianjin 300071, China

^5^Frontiers Science Center for New Organic Matter, College of Chemistry, Nankai University, Tianjin 300071, China

First Author*, Co-Author, Co-Author

*** Correspondence:** Corresponding Author: [kongxianglei@nankai.edu.cn](mailto:kongxianglei@nankai.edu.cn)

# Supplementary Figures

#
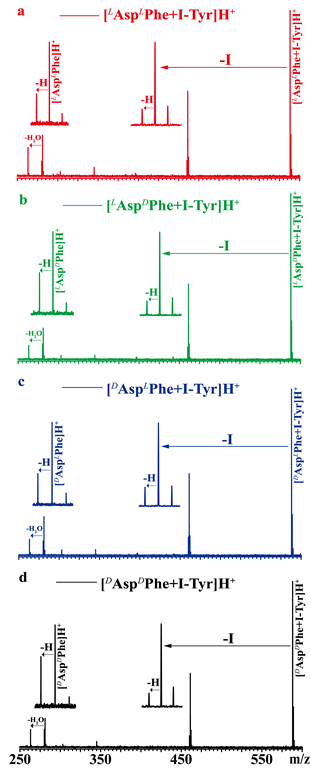


**Supplementary Figure 1.** 280 nm UV photodissociation mass spectra of (a) [*^L^*Asp*^L^*Phe+M_r_]H^+^, (b) [*^L^*Asp*^D^*Phe+M_r_]H^+^, (c) [*^D^*Asp*^L^*Phe+M_r_]H^+^, and (d) [*^D^*Asp*^D^*Phe+M_r_]H^+^, in which the M_r_ indicates the reference molecule of iodo-L-tyrosine.


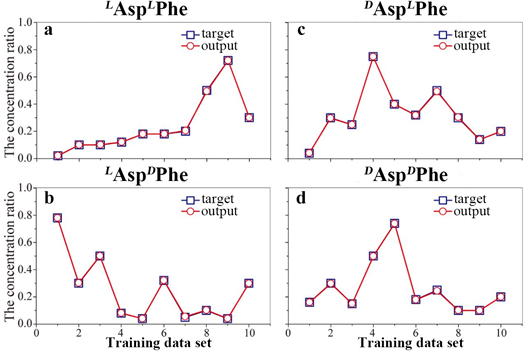


**Supplementary Figure 2.** Trained results of the quantitative analysis of the four samples of *^L/D^*Asp*^L/D^*Phe.


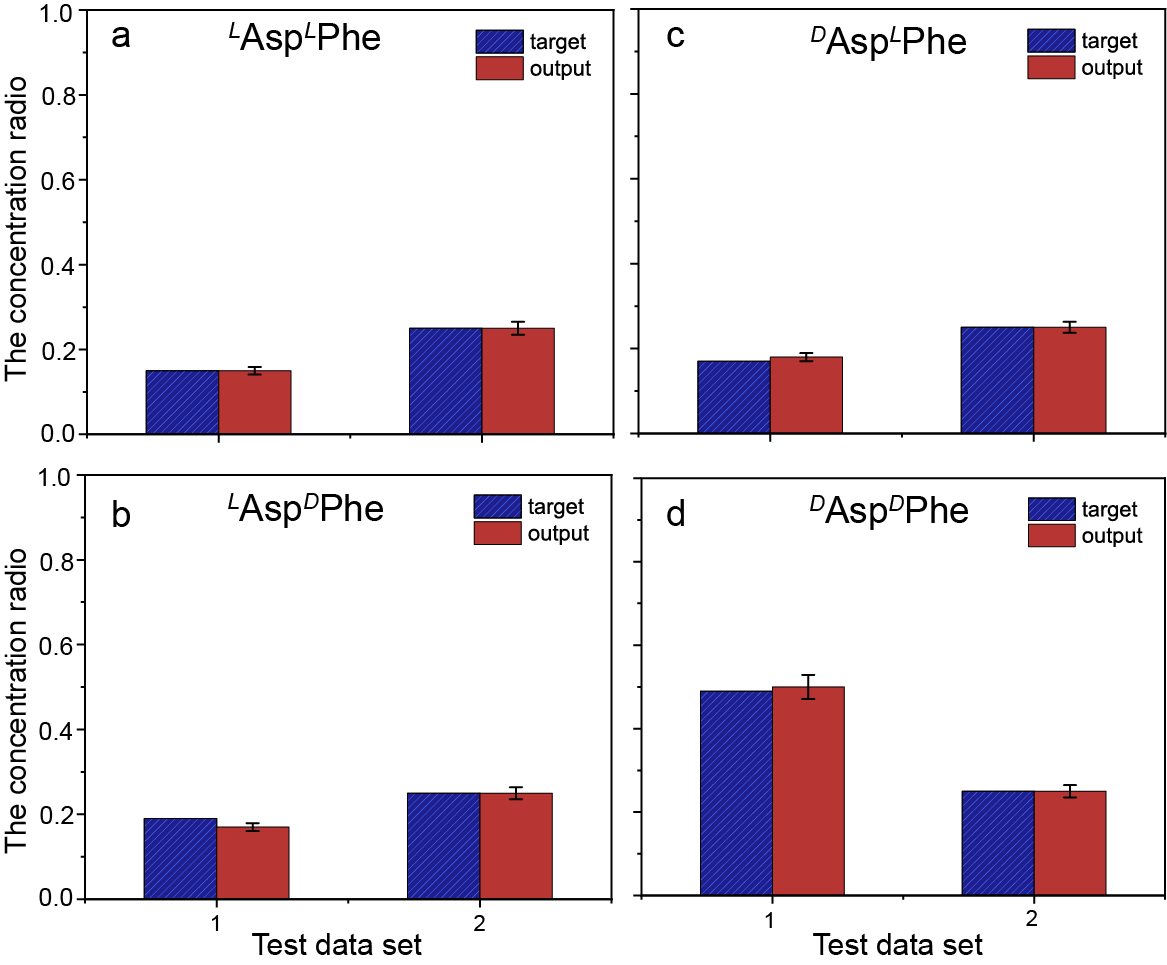


**Supplementary Figure 3.** Comparisons between the true concentration ratios (blue striped columns) and predicted results (red columns) for the quantitative analysis of the four peptides: a) *^L^*Asp*^L^*Phe, b) *^L^*Asp*^D^*Phe, c) *^D^*Asp*^L^*Phe and d) *^D^*Asp/*^D^*Phe. The predictions were performed for four date sets, using the artificial neural network built based on the training set shown in Fig.S2. The standard deviations of the predicted results based on three independent experimental data are less than 6%.
